# The effect of exercise on left ventricular global longitudinal strain

**DOI:** 10.1007/s00421-022-04931-5

**Published:** 2022-03-16

**Authors:** James Murray, Hunter Bennett, Eva Bezak, Rebecca Perry, Terry Boyle

**Affiliations:** 1 Allied Health and Human Performance, University of South Australia, City East Campus, Corner of North Terrace and Frome Rd, Adelaide, SA 5001 Australia; 2 Alliance for Research in Exercise, Nutrition and Activity, University of South Australia, Adelaide, Australia; 3 Cancer Research Institute, University of South Australia, Adelaide, Australia; 4 Australian Centre for Precision Health, University of South Australia Cancer Research Institute, Adelaide, Australia; 5 Department of Physics, University of Adelaide, Adelaide, Australia

**Keywords:** Physical activity, Cardiac function, Cardiovascular imaging

## Abstract

Exercise improves measures of cardiovascular (CV) health and function. But as traditional measures improve gradually, it can be difficult to identify the effectiveness of an exercise intervention in the short-term. Left ventricular global longitudinal strain (LVGLS) is a highly sensitive CV imaging measure that detects signs of myocardial dysfunction prior to more traditional measures, with reductions in LVGLS a strong prognostic indicator of future CV dysfunction and mortality. Due to its sensitivity, LVGLS may offer useful method of tracking the effectiveness of an exercise intervention on CV function in the short-term, providing practitioners useful information to improve patient care in exercise settings. However, the effect of exercise on LVGLS is unclear. This systematic review and meta-analysis aimed to determine the effect exercise has on LVGLS across a range of populations. Included studies assessed LVGLS pre–post an exercise intervention (minimum 2 weeks) in adults 18 years and over, and were published in English from 2000 onwards. Study-level random-effects meta-analyses were performed using Stata (v16.1) to calculate summary standardized mean differences (SMD) and 95% confidence intervals (CI). 39 studies met selection criteria, with 35 included in meta-analyses (1765 participants). In primary analyses, a significant improvement in LVGLS was observed in populations with CV disease (SMD = 0.59; 95% CI 0.16–1.02; *p* = 0.01), however, no significant effect of exercise was observed in CV risk factor and healthy populations. In populations with CV disease, LVGLS could be used as an early biomarker to determine the effectiveness of an exercise regime before changes in other clinical measures are observed.

**Supplementary Information:**

The online version contains supplementary material available at 10.1007/s00421-022-04931-5.

## Introduction

Exercise improves a range of health, fitness and performance indices, spanning a variety of populations (American College of Sports Medicine 2013; Anderson 2016). For cardiovascular (CV) disease and associated risk factors, exercise is a fundamental treatment tool to improve health outcomes and prevent CV events (Sharman et al. 2019; Hordern et al. 2012). Similarly, in healthy populations, exercise is recommended to prevent future chronic health conditions (American College of Sports Medicine 2013).

CV disease is the leading cause of death across Europe (45% of all deaths) (European Heart Network 2017), with an estimated cost to the European Union of €210 billion a year (Timmis et al. 2020). Populations at the greatest risk of CV disease and dysfunction include those with pre-existing CV risk factors such as hypertension (HTN), type 2 diabetes (T2DM), high cholesterol, obesity, history of smoking or alcohol abuse, physical inactivity, and a family history of CV disease (Timmis et al. 2020). Therefore, in these populations, early detection of CV abnormalities and targeted prevention strategies are integral to prevent their progression into CV disease or dysfunction (Eyre et al. 2004).

Global longitudinal strain (GLS) is a highly sensitive CV imaging measure that detects early signs of myocardial dysfunction prior to clinical abnormalities and symptoms arising (D’Elia et al. 2020; Fortuni et al. 2021; Murray et al. 2021). GLS measures myocardial deformation along the longitudinal cardiac axis (Kalam et al. 2014), with reductions in GLS a strong prognostic indicator of future CV dysfunction and mortality (D’Elia et al. 2020; Biering-Sørensen et al. 2017). While GLS is becoming more commonplace in clinical settings to detect sub-clinical changes in myocardial function and identify people at a risk of developing CV dysfunction prior to its onset (Biering-Sørensen et al. 2017; Kaufmann et al. 2019; Negishi et al. 2020), the impact that exercise has on left ventricular (LV) GLS is unclear. Exercise is commonly used to prevent and manage CV disease (Sharman et al. 2019; Hordern et al. 2012), and is known to improve measures of CV health and function (i.e. blood pressure, oxygen consumption (VO2), stroke volume) (Adamopoulos et al. 2014; Lee and Oh 2016). As such, it appears likely that exercise will positively impact LVGLS. Given the sensitivity of LVGLS, it could offer practitioners a viable way to determine the effectiveness of an exercise regime prior to changes in more traditional measures of CV health and function occurring. This could improve patient care by allowing practitioners to adjust exercise prescription based on short-term changes in LVGLS, rather than waiting for long-term changes in CV health and function measures to occur Moreover, if exercise does positively impact LVGLS, it would provide further insight into the mechanistic ability of exercise to prevent clinical CV abnormalities.

Therefore, the aim of this systematic review and meta-analysis was to determine the effect exercise has on LVGLS across a range of healthy, at risk and chronic diseased populations. It was hypothesised that exercise would increase LVGLS in all populations, and to a greater degree in individuals with chronic health conditions that are known to negatively impact LVGLS.

## Methods

The review was conducted in accordance with the Preferred Reporting Items for Systematic Reviews and Meta-Analyses (PRISMA) Statement guidelines (Liberati et al. 2009). The protocol for this review has not been registered with any organisation. A detailed explanation of methodology used can be found in Supplementary Material 2.

### Literature search

Candidate studies published between the years 2000 and 2020 were searched on November 24, 2020 via relevant online databases (Medline, Scopus, eMbase, SPORTDiscus). The following search terms were used: ((exercis* OR train* OR "physical activit*" OR "physical train*" OR "physical rehabilitation" OR "aerobic exercis*" OR "interval train*" OR "resistance train*") AND ("global longitudinal strain" OR "longitudinal strain" OR "GLS" OR "speckle tracking")). The reference lists of included studies were manually searched for additional pertinent articles.

### Selection criteria

**Fig. 1:**
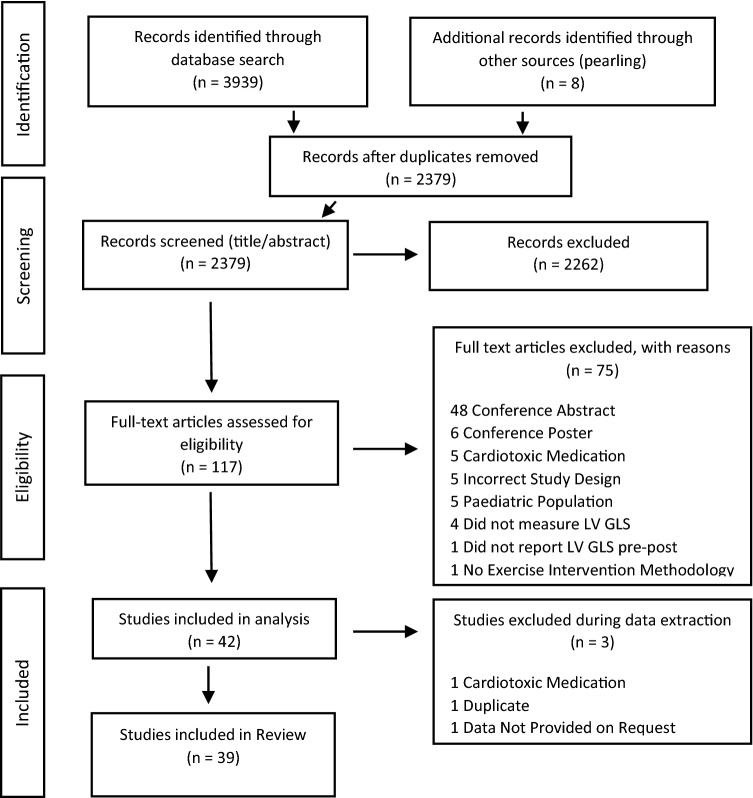
Study identification and selection process

Two authors (JM and HB) independently conducted all database searches, abstract screening, and full text review. Included studies assessed LVGLS before and after an exercise intervention (minimum 2 weeks) in adults aged 18 years and over, and were published in English from 2000 onwards. Whilst still included in the review, papers were excluded from all meta-analyses if the exercise intervention was described inadequately, the same population and data were published across two different papers, or due to missing data (population number, mean, standard deviation). Furthermore, papers that measured LVGLS acutely following a CV event (e.g. acute myocardial infarction, hospitalization for heart failure) were excluded from secondary and exploratory meta-analyses (meta-analyses of intervention group data only), as there was no control group to account for the rapid increase typically seen in LVGLS following an acute event due to revascularization, medication, or other therapies as clinically indicated. The combined database search identified 3939 records, with eight additional records identified via pearling. Following the removal of duplicates, title and abstract screening and full text assessment, 42 studies met the inclusion criteria. A further three studies were excluded during data extraction (Fig. 1), leaving a total of 39 studies for analysis.

### Data extraction and quality assessment

One author (JM) independently extracted all data. Extraction was then cross referenced by (HB), with any discrepancies discussed and resolved. Data were extracted with respect to the following areas: publication demographics, study characteristics, exercise training protocols, outcomes measures. Once the data were extracted, studies were divided into the following health categories to perform primary and secondary meta-analyses: CV disease, CV risk, chronic kidney disease (CKD), healthy, or athletic. Quality assessment of manuscript data was performed independently by two authors (JM and HB) using the QUADAS-2 tool (Whiting et al. 2011). The QUADAS-2 tool was selected as it specifically assesses risk of bias pertaining to the participant selection and testing methodology. Whilst it is predominately used for diagnostic accuracy studies, the domains of this tool still allow for risk of bias in participant selection and measurement to be assessed, whilst also assessing the applicability of each to study to our research question.

### Data analysis

Data referring to intervention characteristics and protocols (age, sample size, health category, exercise intervention) were tabulated and summarised descriptively. Data were presented as mean ± standard deviation (SD) (range). Percentage change in LVGLS from baseline was calculated for each individual study using the following formula: [(post LVGLS—pre LVGLS)/pre LVGLS] × 100. These results were then summarised descriptively.

Both primary and secondary analyses were performed in this review. Primary meta-analyses included randomised control trials (RCTs), non-randomised control trials (N-RCTs) and randomised cross-over studies that compared outcomes between one or more intervention arms to a standard (non-exercising) control arm. In studies with two intervention arms (i.e. high intensity interval training vs moderate intensity continuous training), each arm was included separately in the meta-analysis, with the control group population (n) halved. Secondary and exploratory meta-analyses included data from the exercise groups of RCT’s, N-RCT’s and cross-over studies, and data from studies with intervention arms only (single group pre-post studies).

All meta-analyses were performed using Stata (v16.1, StataCorp, TX, USA). A random-effects model (restricted maximum likelihood) was used for all meta-analyses. Random-effects meta-analyses were conducted due to the different characteristics of the interventions and study population in the eligible studies (Borenstein et al. 2010). Standardized mean difference (SMD) was calculated using Cohen’s d statistic (Cohen 2013) with 95% confidence intervals (CI) for all meta-analyses. SMD was categorised as small (0.2), moderate (0.5) and large (0.8) (Cohen 2013). Heterogeneity was calculated using the *I*^2^ statistic. *I*^2^ values of 25%, 50% and 75% were considered to represent low, moderate, and high heterogeneity, respectively. *P* < 0.05 indicated statistical significance. Further details regarding the calculation of SMD for primary, secondary, and exploratory meta-analysis is outlined in Supplementary Material 2.

Publication bias of primary and secondary meta-analyses were evaluated using funnel plots and Eggers tests (Egger et al. 1997).

## Results

The quality appraisal ratings of the 39 included papers are presented in Supplementary Material 1, Table 1. Of the 273 ratings given across 39 studies, 88% were low for risk of bias and concerns regarding applicability. Risk of bias was most apparent in the patient selection domain due to unclear recruitment procedures and the selection of non-random samples. Applicability concerns were present in only one study (Isbel et al. 2013), as measurement procedures were not outlined.

**Table 1:** Summary of relative percentage change in LVGLS from baseline per health category (data presented in number of studies/study groups)

Population	*n*	Relative change in LVGLS from baseline (number of studies)							
		< 0–5%		5–10%		10–15%		> 15%	
		EX	CON	EX	CON	EX	CON	EX	CON
*Primary analysis*									
Cardiovascular disease	4	1	3	1	1	–	–	2	-
Cardiovascular risk	5	3	1	1		–	1	2	2
Healthy	5	5	3	–	2	–	–	–	
*Secondary analysis*									
Cardiovascular disease	7	5		1		1		–	
Cardiovascular risk	9	5		2		1		1	
Renal	3	1		–		1		1	
Healthy	14	12		2		–		–	
Athletic	6	5		1		–		–	

*n* number of studies, *EX* exercising group, *CON* control group.

The intervention characteristics and protocols of the 39 published studies are presented in Supplementary Material 1, Table 2. Research designs consisted of single cohort observations (43.6%), randomised control trials (41%), non-randomised control trials (12.8%), and randomised cross-over trials (2.6%). A total of 1,972 participants were included across the 39 studies, with 1,491 participants exposed to an exercise intervention. Health categories consisted of healthy (28.2%), CV disease (25.6%), CV risk factor (25.6%), athletes (12.8%) and CKD (7.7%). With respect to training protocols, the average exercise intervention spanned 20.7 ± 19.9 (range 2–104) weeks with 3.9 ± 1.5 (range 2–8) sessions per week. Over half (53.8%) of the studies prescribed aerobic exercise only (either continuous, interval or both), 43.6% prescribed a combination of aerobic and resistance training, with 2.6% prescribing resistance training only. All included studies were published between 2009 and 2020. Information regarding the measurement of LVGLS, including the views, machine and analysis software used, as well as the number of images excluded due to poor image quality in each study is reported in Supplementary Material 1, Table 3.

The following papers were excluded from all meta-analyses due to the reasons outlined in Section Selection Criteria (Acar et al. 2015; Ofstad et al. 2014; Oxborough et al. 2019; Santoso et al. 2019). Therefore, 35 studies with a total of 1765 participants were included in meta-analyses. The following papers were excluded from secondary and exploratory meta-analyses due to the reasons outlined in Sect. Selection Criteria (Malfatto et al. 2017; McGregor et al. 2018; Trachsel et al. 2019; Xu et al. 2016).

## Primary meta-analyses

**Fig. 2:**
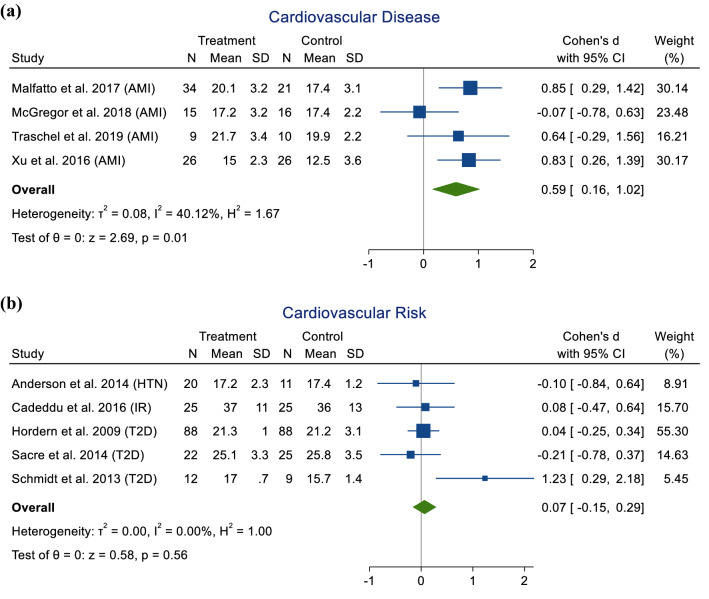
Meta-analysis of randomised control trials, non-randomised control trials and randomised cross-over trials investigating the effect of exercise on LVGLS in cardiovascular disease (**a**) and cardiovascular risk factor (**b**) populations. *AMI* acute myocardial infarction, *HTN* hypertension, *IR* insulin resistance, *T2D* type 2 diabetes, *N* number of participants, *SD* standard deviation, *CI* confidence intervals

In populations with CV disease, a moderate effect of exercise was observed compared to non-exercising controls (SMD = 0.59; 95% CI 0.16–1.02; *p* = 0.01), with moderate heterogeneity (*I*^2^ = 40.12%) (Fig. 2a). There was no significant effect of exercise in CV risk (SMD = 0.07; 95% CI − 0.15–0.29; *p* = 0.56; *I*^2^ = 0.00%) (Fig. 2b) or healthy populations (SMD = − 0.20; 95% CI − 0.73–0.33; *p* = 0.45; *I*^2^ = 59.08%) compared to non-exercising controls (Supplementary Material 1, Fig. 12).

### Secondary meta-analyses

**Fig. 3:**
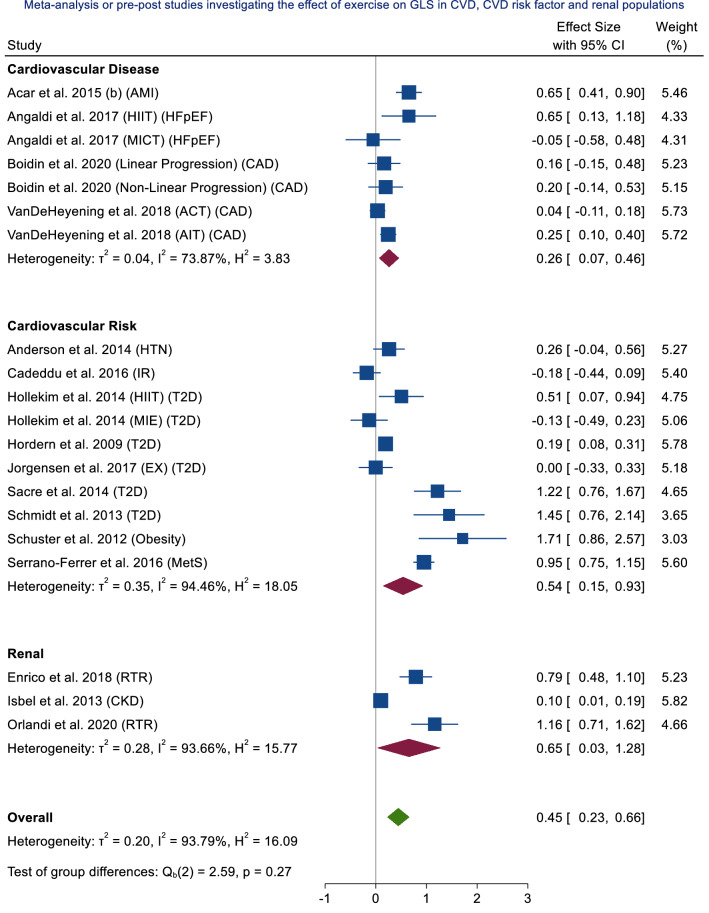
Meta-analysis of exercise data from randomised control trials, non-randomised control trials, randomised cross-over trials and single group pre-post studies investigating the effect of exercise on LVGLS in cardiovascular disease (CVD), cardiovascular (CV) risk factor and chronic kidney disease (CKD) populations. *AMI* acute myocardial infarction, *HIIT* high intensity interval training, *HFpEF* heart failure preserved ejection fraction, *MICT* moderate intensity continuous training, *CAD* coronary artery disease, *ACT* aerobic continuous training, *AIT* aerobic interval training, *HTN* hypertension, *IR* insulin resistance, *T2D* type 2 diabetes, *EX* exercise, *MetS* metabolic syndrome, *RTR* renal transplant recipient, *CKD* chronic kidney disease, *CI* confidence intervals

Across all clinical populations (CV disease, CV risk, CKD), a SMD of 0.45 was observed (95% CI 0.23–0.66), with high heterogeneity (*I*^2^ = 93.79%) (Fig. 3). In populations with CV disease alone, a small effect was observed (SMD = 0.26; 95% CI 0.07–0.46; *p* = 0.01), with high heterogeneity (*I*^2^ = 73.87%) (Supplementary Material 1, Fig. 13). In populations with CV risk factors, a moderate effect was observed (SMD = 0.54; 95% CI 0.15–0.93; *p* = 0.01), with high heterogeneity (*I*^2^ = 94.46%) (Supplementary Material 1, Fig. 14). In CKD populations alone, a moderate effect was observed (SMD = 0.65; 95% CI 0.03–1.28; *p* = 0.04), with high heterogeneity (*I*^2^ = 93.66%) (Supplementary Material 1, Fig. 15).

**Fig. 4:**
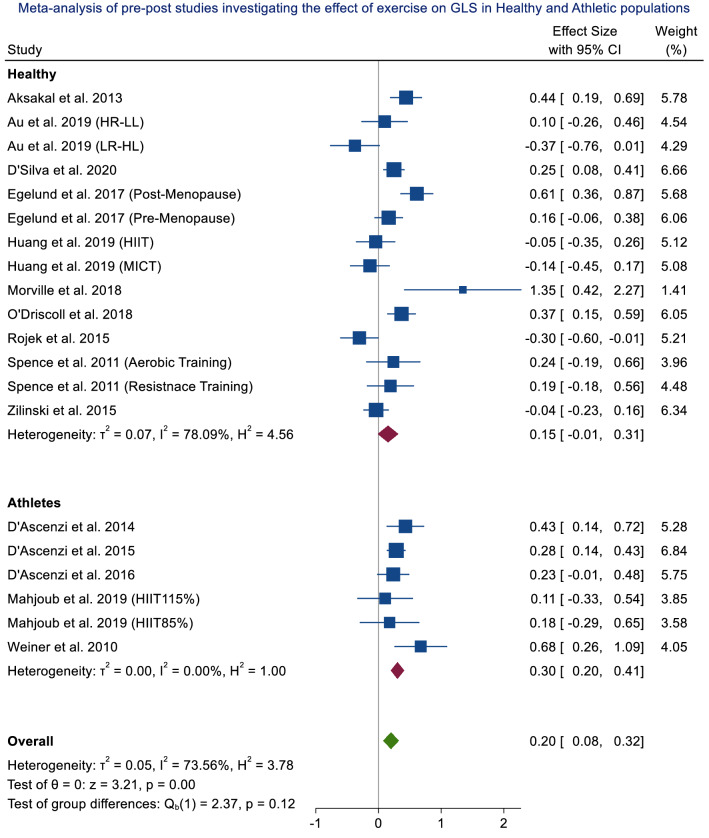
Meta-analysis of exercise data from randomised control trials, non-randomised control trials, randomised cross-over trials and single group pre-post studies investigating the effect of exercise on LVGLS in healthy and athletic populations. *HR-LL* high repetitions, low load, *LR-HL* low repetitions, high load, *HIIT* high intensity interval training, *MICT* moderate intensity continuous training, *CI* confidence intervals

Across non-clinical populations (healthy and athletic), a SMD of 0.20 was observed (95% CI, 0.08–0.32), with high heterogeneity (*I*^2^ = 73.56%) (Fig. 4). In athletic populations alone, a small effect was observed (SMD = 0.30; 95% CI 0.20–0.41; *p* =  < 0.001), with no heterogeneity (*I*^2^ = 0.00%) (Supplementary Material 1, Fig. 17). There was no significant effect of exercise in healthy populations alone (SMD = 0.15; 95% CI 0.01–0.31; *p* = 0.06; *I*^2^ = 78.09%) (Supplementary Material 1, Fig. 16).

A numerical summary of results from primary and secondary meta-analyses conducted across each health category are presented in Supplementary Material 1, Table 4.

### Sub-group exploratory analysis

All sub-group meta-analyse forest plots can be found in Supplementary Material 1 (Figs. 9–11), with a numerical summary presented in Supplementary Material 1, Table 5.

There was no significant difference between exercise intervention lengths on LVGLS (between group difference *p* = 0.06), with short (SMD = 0.38; 95% CI, 0.08–0.69; *p* = 0.01), moderate (SMD = 0.17; 95% CI, 0.05–0.29; *p* =  < 0.001), and long (SMD = 0.50; 95% CI, 0.23–0.76; *p* =  < 0.001) interventions all demonstrating significant, positive effects.

Similarly, there was no difference (*p* = 0.38) between aerobic only (SMD = 0.29; 95% CI 0.15–0.43; *p* =  < 0.001) and combined aerobic and resistance (SMD = 0.42; 95% CI 0.17–0.66; *p* =  < 0.001) exercise interventions on LVGLS, or between aerobic continuous (SMD = 0.25; 95% CI 0.03–0.47; *p* = 0.01) and aerobic interval (SMD = 0.34; 95% CI 0.18–0.50; *p* =  < 0.001) based interventions (*p* = 0.50).

### Publication bias

Funnel plots and Eggers tests suggested evidence of publication bias in primary meta-analyses of healthy populations (*p* = 0.0039), and secondary meta-analyses of CV risk (*p* = 0.0099) and CKD (*p* =  < 0.001) populations (Supplementary Material 1, Figs. 3, 5, 6).

### Clinical significance

Table 1 presents a summary of the relative percentage change in LVGLS from baseline per health category. A 10% relative change from baseline in LVGLS (either negative or positive) is considered clinically significant (Yang et al. 2018). Across primary meta-analyses, two CV disease studies reported clinically significant increases in LVGLS following exercise (Malfatto et al. 2017; Xu et al. 2016). Similarly, two CV risk studies reported clinically significant increases in LVGLS following exercise (Cadeddu et al. 2016; Sacre et al. 2014), however, clinically significant increases were also seen in three non-exercising control groups (Cadeddu et al. 2016; Sacre et al. 2014; Andersen et al. 2014). Five studies included in secondary meta-analyses observed clinically significant increases in LVGLS following exercise (Sacre et al. 2014; Angadi et al. 2017; Enrico et al. 2018; Orlandi et al. 2020; Serrano-Ferrer et al. 2016).

## Discussion

This study is the first to review and analyse the effect of exercise on LVGLS across a range of CV diseased, CV risk, healthy, and athletic populations. To provide a comprehensive evaluation of the literature, both primary and secondary analyses were performed. Primary meta-analyses included RCTs, N-RCTs and randomised cross-over studies that compared outcomes between one or more intervention arms to a standard (non-exercising) control arm, with secondary meta-analyses including data from the exercise groups of RCT’s, N-RCT’s and cross-over studies, and data from studies with intervention arms only (single group pre–post studies). In populations with overt CV disease, the SMD from primary analyses indicates that exercise significantly increased LVGLS. These same findings were not observed in primary analyses with healthy populations and populations at risk of developing overt CV disease, with meta-analyses finding no significant effect of exercise on LVGLS. In secondary meta-analyses of pre–post studies and the intervention arm of RCTs, exercise significantly increased LVGLS in populations with overt CV disease, at risk of developing CV disease, with CKD, and athletes.

Given the known benefits of exercise on CV health and function (Sharman et al. 2019; Hordern et al. 2012), it was hypothesised that exercise would increase LVGLS (being a measure of myocardial function). In our primary meta-analyses, this hypothesis was upheld for populations with overt CV disease. Cardiac rehabilitation (in the form of aerobic exercise) following acute myocardial infarction is used extensively to promote myocardial recovery, improve cardiorespiratory fitness, and prevent secondary events occurring in such populations (Lavie and Milani 2000). Findings from this meta-analysis may provide mechanistic insight into how cardiac rehabilitation (aerobic exercise) may improve myocardial function in this specific population (overt CV disease), by increasing LVGLS. As reductions in LVGLS is a marker of early myocardial dysfunction and mortality, the increases observed further emphasise the importance of regular exercise in populations with overt CV disease to promote myocardial function and prevent future CV abnormalities. Interestingly, this same increase was not observed in primary analyses of populations at risk of developing CV disease. In those with HTN or T2DM (CV disease risk factors), regular exercise has been shown to reduce blood pressure and blood sugar, and in some cases even replace medication to manage these risks (Park et al. 2021). It is possible that medication in certain exercising participants within these studies may have ceased during the intervention as exercise may have improved blood pressure or blood sugar levels. Therefore, while change in LVGLS between exercise and control groups was not statistically different, the potential reductions in medication dose in exercising participants would carry noteworthy clinical importance. However, as these studies did not report participant medication information, this cannot be stated with certainty. The similar non-significant change observed in primary analyses of healthy populations may be attributed to the inclusion of two resistance training groups. Few studies (Au et al. 2019; Spence et al. 2011) have investigated the effect of resistance training on LVGLS, with results of these studies finding no effect and/or a negative effect in comparison to control. Given that resistance training is performed primarily to promote peripheral adaptions (rather than central adaptations), the non-effect observed in these studies could be easily explained. More research is required to understand the effect resistance training has on LVGLS. However, given results across other populations, and that LVGLS is measure of myocardial function, it is likely that aerobic exercise is the preferred modality to elicit increases in LVGLS. It is important to note that as LVGLS did increase in response to exercise in populations with overt CV disease, it may offer a viable, early biomarker to determine the effectiveness of an exercise intervention in this population. If changes in LVGLS precede changes in other measures of CV health and function, it could be measured regularly throughout the duration of an exercise program to ensure it is achieving the desired outcomes. As such, in hospital and/or multidisciplinary settings where cardiac sonography expertise and equipment is readily available, the regular measurement of LVGLS in CV patients could be recommended in addition to other traditional measures of CV health and function. Future research should explore the time course of LVGLS change in response to exercise relative to other CV risk factors, identifying its practical utility in this setting. Furthermore, as reductions in LVGLS are predictive of CV dysfunction (Biering-Sørensen et al. 2017), future research should explore whether exercise-induced improvements in LVGLS are associated with improvements in other measures of CV health and function (i.e. blood pressure, VO2, stroke volume).

Secondary meta-analyses demonstrated statistically significant increases in LVGLS following exercise in CV diseased, CV risk, CKD, and athletic populations. Whilst conclusions cannot be drawn from these analyses (due to the many confounding factors that cannot be accounted for without a control group), these findings do suggest exercise may be used as a therapeutic intervention to increase LVGLS across each population sub-group. Interestingly, secondary analyses of populations at risk of developing CV disease saw a SMD of 0.54, despite primary analyses suggesting no effect of exercise on LVGLS in CV risk populations. Given the limited number of RCTs across this health category, the positive increase observed in pre-post studies, and the importance of preventing progression into overt CV disease for this population, there is merit for further RCTs to investigate the effect of exercise on LVGLS in populations at risk of CV disease.

Exploratory meta-analyses indicated that there was no specific intervention length or exercise modality that impacted the change in LVGLS greater than another. Although minor differences in SMD’s between different groups were observed (Supplementary Material 1, Table 4), all intervention lengths and exercise modalities significantly increased LVGLS, with no statistically significant differences observed between any groups. Although not conclusive, this analysis suggests that any aerobic exercise performed for a minimum of 2 weeks may be sufficient to increase LVGLS. It must be noted that the number of sessions per week were not accounted for in this analysis, and as such, the total volume of exercise performed may have overlapped between intervention length categories (i.e. a 12 week intervention with 2 sessions a week has the same exercise volume as an intervention lasting 6 weeks with 4 sessions per week). However, these findings suggest that > 2 weeks of exercise may increase LVGLS, highlighting its effectiveness to practitioners and patients alike.

Nine exercising groups reported clinically significant increases in LVGLS (four primary studies, five secondary studies) (Malfatto et al. 2017; Xu et al. 2016; Cadeddu et al. 2016; Sacre et al. 2014; Andersen et al. 2014; Angadi et al. 2017; Enrico et al. 2018; Orlandi et al. 2020; Serrano-Ferrer et al. 2016) (Table 1). However, clinically significant increases were also seen in three non-exercising control groups (Cadeddu et al. 2016; Sacre et al. 2014; Andersen et al. 2014), making this finding difficult to interpret. Of further interest, no exercising group included in primary or secondary meta-analyses observed a clinically significant reduction in LVGLS, suggesting exercise does not negatively impact LVGLS.

### Strengths, limitations and future research

The high number of included studies is a key strength of this review. Further strengths include systematic database searches, with hand searching of reference lists of eligible studies and screening and data extraction confirmed for consistency by two, and if required three, independent authors. Additionally, only including trials with an active control arm in the primary analysis, and excluding studies that contained participants acutely following a CV event (e.g. acute myocardial infarction, hospitalization for heart failure) from secondary and exploratory meta-analyses, can increase confidence the results likely indicate the effect of exercise on LVGLS. Due to the inability to access individual participant data, this meta-analysis was performed at a study level. The lack of reporting of participant level data also prevented an evaluation of how exercise impacted individual changes in participant health status or medication regime. As a result, it is also unclear if changes in medication at a participant level impacted upon the changes in LVGLS reported in included studies. However, this is likely mitigated by only including trials with an active control arm in the primary analysis. This would have provided more context on the effect of exercise on LVGLS, with respect to potential changes in medication dose. As LVGLS was not a primary outcome measure in all studies, certain groups included in primary meta-analyses were not balanced for LVGLS at baseline. With baseline LVGLS likely to be strongly correlated with post-intervention LVGLS, future studies should consider pre-specifying that baseline LVGLS will be included as a covariate when testing for differences in post-intervention-LVGLS (Vickers and Altman 2001). Information regarding the views used to measure LVGLS is reported in Supplementary Material 1, Table 3. Image quality and how/if any images were excluded from analysis was not well reported in included papers, however, where known has been reported in Supplementary Material 1, Table 3. Different machines and software were used to measure LVGLS across different studies, which may also be considered a limitation. Despite the fact that there were different methods and software used or not reported in some studies the same methodology was used consistently within each study making the results valid for this analysis. Future studies must report all variables relating to the measurement of LVGLS to ensure consistency in the literature and prevent bias across individual studies. Results of primary meta-analyses in healthy populations, and secondary meta-analyses in CV risk and CKD populations must be treated with some caution due to the possibility of publication bias, as indicated by significant Eggers Test results (*p* = 0.0039, *p* = 0.0099, *p* =  < 0.001, respectively). The intensity of exercise prescribed was poorly reported amongst studies included in this review. As such, no analysis on the effect of exercise intensity on LVGLS was performed, and this relationship remains unknown. Future research must address the methodological limitations in study designs and reporting discussed above.

## Conclusion

In populations with overt CV disease, exercise significantly increased LVGLS, suggesting it could be used as an early biomarker to determine the effectiveness of an exercise regime before changes in other clinical measures are observed in this population. Similar findings were not observed in primary meta-analyses of CV risk and healthy populations. Secondary meta-analyses suggest exercise may be used as a therapeutic intervention to increase LVGLS in CV diseased, CV risk, CKD, and athletic populations. Given results of secondary meta-analyses, the importance of prevention of CV disease, and the limitations in current study designs, there is merit for further RCTs to investigate the effect of exercise on LVGLS in at risk CV populations, whilst addressing the methodological limitations that currently exist.

## Supplementary Information

Below is the link to the electronic supplementary material.

Supplementary file1 (DOCX 208 KB)

Supplementary file2 (DOCX 23 KB)
