# Supplementary material for: The effect of exercise on left ventricular global longitudinal strain
Source: Eur J Appl Physiol. 2022 Mar 16;122(6):1397–408. doi: 10.1007/s00421-022-04931-5 (PMC9132819; doi:10.1007/s00421-022-04931-5)
Supplement: Supplementary file 1 — Supplementary file1 (DOCX 208 KB) [file 421_2022_4931_MOESM1_ESM.docx]

**Supplementary Material**

***List of Contents (in order of appearance):***

**Table 1**. Risk of bias and applicability concerns for individual studies in each domain, according to the QUADAS-2 tool.

**Figure 1.** Funnel plot of randomised control trials, non-randomised control trials and randomised crossover trials investigating the effect of exercise on LVGLS in populations with cardiovascular disease.

**Figure 2.** Funnel plot of randomised control trials, non-randomised control trials and randomised crossover trials investigating the effect of exercise on LVGLS in populations at risk of developing cardiovascular disease

**Figure 3.** Funnel plot of randomised control trials, non-randomised control trials and randomised crossover trials investigating the effect of exercise on LVGLS in healthy populations.

**Figure 4.** Funnel plot of exercise data from randomised control trials, non-randomised control trials, randomised cross over trials and single group pre-post studies investigating the effect of exercise on LVGLS in cardiovascular disease populations.

**Figure 5.** Funnel plot of exercise data from randomised control trials, non-randomised control trials, randomised cross over trials and single group pre-post studies investigating the effect of exercise on LVGLS in populations at risk of developing cardiovascular disease.

**Figure 6.** Funnel plot of exercise data from randomised control trials, non-randomised control trials, randomised cross over trials and single group pre-post studies investigating the effect of exercise on LVGLS in chronic kidney disease populations. Eggers test p = <0.001

**Figure 7.** Funnel plot of exercise data from randomised control trials, non-randomised control trials, randomised cross over trials and single group pre-post studies investigating the effect of exercise on LVGLS in healthy populations.

**Figure 8.** Funnel plot of exercise data from randomised control trials, non-randomised control trials, randomised cross over trials and single group pre-post studies investigating the effect of exercise on LVGLS in athletic populations.

**Table 2.** Intervention characteristics and protocols

**Table 3.** Methods and reporting of strain analysis.

**Figure 9.** Effect of exercise intervention length on LVGLS using exercise data from randomised control trials, non-randomised control trials, randomised cross over trials and single group pre-post studies.

**Figure 10.** Effect of exercise modality on LVGLS using exercise data from randomised control trials, non-randomised control trials, randomised cross over trials and single group pre-post studies – Comparison between aerobic training only vs a combination of aerobic and resistance training.

**Figure 11.** Effect of exercise modality on LVGLS using exercise data from randomised control trials, non-randomised control trials, randomised cross over trials and single group pre-post studies – Comparison between aerobic continuous training vs aerobic interval training.

**Figure 12.** Figure 4. Randomised control trials, non-randomised control trials and randomised crossover trials investigating the effect of exercise on LVGLS in healthy populations alone.

**Figure 13.** Exercise data from randomised control trials, non-randomised control trials, randomised cross over trials and single group pre-post studies investigating the effect of exercise on LVGLS in cardiovascular disease populations alone.

**Figure 14.** Exercise data from randomised control trials, non-randomised control trials, randomised cross over trials and single group pre-post studies investigating the effect of exercise on LVGLS in populations with cardiovascular risk factors alone.

**Figure 15.** Single group pre-post studies investigating the effect of exercise on LVGLS in chronic kidney disease populations alone.

**Figure 16.** Exercise data from randomised control trials, non-randomised control trials, randomised cross over trials and single group pre-post studies investigating the effect of exercise on LVGLS in healthy populations alone.

**Figure 17.** Single group pre-post studies investigating the effect of exercise on LVGLS in athletic populations alone.

**Table 4.** Summary of meta-analysis outcomes per health category.

**Table 5**. Summary of exploratory meta-analyses using pre-post study data.

**Reference List.** Reference list of included studies.

**Table 1.** Risk of bias and applicability concerns for individual studies in each domain, according to the QUADAS-2 tool.

|  |  | **Risk of Bias** | | | | | **Applicability Concerns** | | |
| --- | --- | --- | --- | --- | --- | --- | --- | --- | --- |
| **Author** | **Patient Selection** | | **Index test** | **Reference Standard** | **Flow and Timing** | **Patient selection** | | **Index test** | **Reference Standard** |
| Acar et al. 2015 | U | | L | L | L | L | | L | L |
| Acar et al. 2015 (b) | U | | L | L | L | L | | L | L |
| Aksakal et al. 2013 | L | | L | L | L | L | | L | L |
| Anderson et al. 2014 | L | | L | L | L | L | | L | L |
| Angadi et al. 2017 | L | | L | L | L | L | | L | L |
| Au et al. 2019 | L | | L | L | L | L | | L | L |
| Boidin et al. 2020 | L | | L | L | L | L | | L | L |
| Cadeddu et al. 2016 | U | | L | L | L | L | | L | L |
| D'Ascenzi et al. 2014 | L | | L | L | L | L | | L | L |
| D'Ascenzi et al. 2015 | L | | L | L | L | L | | L | L |
| D'Ascenzi et al. 2016 | L | | L | L | L | L | | L | L |
| D'Silva et al. 2020 | L | | L | L | L | L | | L | L |
| Egulund et al. 2017 | L | | L | L | L | L | | L | L |
| Enrico et al. 2018 | U | | L | L | L | L | | L | L |
| Hollekim et al. 2014 | L | | U | U | L | L | | L | L |
| Hordern et al. 2009 | L | | L | U | L | L | | L | L |
| Huang et al. 2019 | L | | L | L | L | L | | L | L |
| Isbel et al. 2013 | U | | U | U | L | L | | U | U |
| Jorgensen et al. 2017 | U | | L | L | L | L | | L | L |
| Mahjoub et al. 2019 | U | | L | L | L | L | | L | L |
| Malfatto et al. 2017 | H | | L | L | L | L | | L | L |
| McGregor et al. 2018 | U | | L | L | L | L | | L | L |
| Morville et al. 2018 | H | | L | L | H | L | | L | L |
| O'Driscoll et al. 2018 | L | | L | L | L | L | | L | L |
| Ofstad et al. 2014 | L | | L | L | U | L | | L | L |
| Orlandi et al. 2020 | L | | L | L | L | L | | L | L |
| Oxborough et al. 2019 | L | | L | L | L | L | | L | L |
| Rojek et al. 2015 | H | | L | L | L | L | | L | L |
| Scare et al. 2014 | H | | L | L | L | L | | L | L |
| Santoso et al 2019 | L | | L | L | L | L | | L | L |
| Schmidt et al. 2013 | L | | L | L | L | L | | L | L |
| Schuster et al. 2012 | U | | L | L | L | L | | L | L |
| Serrano-Ferrer et al. 2016 | U | | L | L | L | L | | L | L |
| Spence et al. 2011 | L | | L | L | L | L | | L | L |
| Trachsel et al. 2019 | L | | L | L | L | L | | L | L |
| VanDeHeyning et al. 2018 | L | | L | L | L | L | | L | L |
| Weiner et al. 2010 | U | | L | L | L | L | | L | L |
| Xu et al. 2016 | L | | L | L | L | L | | L | L |
| Zilinski et al. 2015 | L | | L | L | L | L | | L | L |

Abbreviations: H - high; L - low; U - unclear.

**Figure 1.** Funnel plot of randomised control trials, non-randomised control trials and randomised crossover trials investigating the effect of exercise on LVGLS in populations with cardiovascular disease. Eggers test p = 0.5339.

**Figure 2.** Funnel plot of randomised control trials, non-randomised control trials and randomised crossover trials investigating the effect of exercise on LVGLS in populations at risk of developing cardiovascular disease. Eggers test p = 0.2315.

**Figure 3.** Funnel plot of randomised control trials, non-randomised control trials and randomised crossover trials investigating the effect of exercise on LVGLS in healthy populations. Eggers test p = 0.0039.

**Figure 4.** Funnel plot of exercise data from randomised control trials, non-randomised control trials, randomised cross over trials and single group pre-post studies investigating the effect of exercise on LVGLS in cardiovascular disease populations. Eggers test p = 0.7353.

**Figure 5.** Funnel plot of exercise data from randomised control trials, non-randomised control trials, randomised cross over trials and single group pre-post studies investigating the effect of exercise on LVGLS in populations at risk of developing cardiovascular disease. Eggers test p = 0.0099.

**Figure 6.** Funnel plot of exercise data from randomised control trials, non-randomised control trials, randomised cross over trials and single group pre-post studies investigating the effect of exercise on LVGLS in chronic kidney disease populations. Eggers test p = <0.001


**Figure 7.** Funnel plot of exercise data from randomised control trials, non-randomised control trials, randomised cross over trials and single group pre-post studies investigating the effect of exercise on LVGLS in healthy populations. Eggers test p = 0.2577.

**Figure 8.** Funnel plot of exercise data from randomised control trials, non-randomised control trials, randomised cross over trials and single group pre-post studies investigating the effect of exercise on LVGLS in athletic populations. Eggers test p = 0.7008.

**Table 2.** Intervention characteristics and protocols.

| **Author** | **Study Design** | **Health Category** | **Population details** | **Group** | **n** | **Age [(yrs.). Mean ± SD]** | **Exercise Modality** | **Length of Intervention (wks)** | **Frequency (sessions per wk)** | **Session Duration (mins)** | **Exercise Intensity** | **Relative Change in GLS from Baseline (%)** |
| --- | --- | --- | --- | --- | --- | --- | --- | --- | --- | --- | --- | --- |
| Acar et al. 2015 | Non-RCT | Cardiovascular Disease | Patients following Acute MI | EX | 27 | 57 ± nr | Aerobic (CR) | 12 | 3-4 | 40 | nr | 30.77 |
|  |  |  |  | CON | 27 | 58 ± nr |  |  |  |  |  | nr |
| Acar et al. 2015 (b) | Cohort pre-post | Cardiovascular Disease | Patients following Acute MI | EX | 42 | 57.1 ± 7 | Aerobic (CR) | 6 | 5 | 30-60 | 13-15 RPE | 9.27 |
| Aksakal et al. 2013 | Cohort pre-post | Healthy | Healthy sedentary young men | EX | 34 | 21.6 ± 2 | Aerobic | 24 | nr | 1-4hrs per day | 11-20 RPE | 3.03 |
| Anderson et al. 2014 | Cohort pre-post | Cardiovascular Risk | Sedentary males with mild to moderate hypertension | EX | 20 | 45.8 ± 7.2 | Aerobic | 24 | 2 | 60 | nr | 3.61 |
|  |  |  |  | CON | 11 | 46.9 ± 7.6 |  |  |  |  |  | 12.26 |
| Angaldi et al. 2017 | RCT | Cardiovascular Disease | Patients with HFpEF | EX (HIIT | 9 | 69 ± 6.1 | Aerobic (HIIT vs MICT) | 4 | 3 | HIIT: 28 | HIIT: 85-90% peak HR | 13.29 |
|  |  |  |  | EX (MICT) | 6 | 71.5 ± 11.7 |  |  |  | MICT: 30 | MICT: 75% peak HR | -1.25 |
| Au et al. 2019 | RCT | Healthy | Men with a history of recreational resistance training | EX (HR-LL) | 13 | 23 ± 3 | Resistance | 12 | 4 | nr | HR-LL: 3x20-25 @ 30-50% 1RM | 1.24 |
|  |  |  |  | EX (LR-HL) | 13 | 23 ± 2 |  |  |  |  | LR-HL: 3x8-12 @ 75-90% 1RM | -4.71 |
|  |  |  |  | CON | 14 | 24 ± 2 |  |  |  |  |  | 8.07 |
| Boidin et al. 2020 | RCT | Cardiovascular Disease | Adults with stable CAD | EX (LP) | 18 | 64 ± 11 | Aerobic + Resistance | 12 | 3 | 20-60 | 50-100% peak power output | 2.89 |
|  |  |  |  | EX (NLP) | 16 | 66 ± 5 |  |  |  |  |  | 3.37 |
| Cadeddu et al. 2016 | Non-RCT | Cardiovascular Risk | Patients with impaired glucose tolerance and/ or impaired fasting glucose | EX (+metformin) | 25 | 45.5 ± 12 | Aerobic | 12 | 4 | 60 | Anaerobic threshold | 68.18 |
|  |  |  |  | EX (only) | 25 | 46 ± 12 |  |  |  |  |  | -8.33 |
|  |  |  |  | CON | 25 | 47.1 ±11 |  |  |  |  |  | 33.33 |
| D'Ascenzi et al. 2014 | RCT | Athletes | Female elite volleyball players | EX | 24 | 24.9 ± 4.1 | Aerobic | 16 | 8 | 120 | nr | 4.06 |
|  |  |  |  | CON | 24 | 24.4 ± 3.2 |  |  |  |  |  | nr |
| D'Ascenzi et al. 2015 | Cohort pre-post | Athletes | Professional athletes engaged in soccer, basketball and volleyball | EX | 91 | 23 ± 6 | Aerobic + Resistance | 18 | Aerobic: nr  Resistance: 2 | nr | Aerobic:70-95% HRmax  Resistance: Moderate intensity | 2.97 |
| D'Ascenzi et al. 2016 | Cohort pre-post | Athletes | Elite athletes practicing basketball and volleyball | EX | 29 | 20.9 ± 6.7 | Aerobic + Resistance | 26 | Aerobic: nr  Resistance: 2 | nr | Aerobic:70-95% HRmax  Resistance: Moderate intensity | 3.59 |
| D'Silva et al. 2020 | Cohort pre-post | Healthy | Young adults aged 18-35 | EX | 68 | 29.5 ± 3.2 | Aerobic | 17 | 3 | 20-180 | nr | 3.89 |
| Egelund et al. 2017 | Cohort pre-post | Healthy | Women either pre- or post-menopause | EX (PRE-M) | 36 | 49.4 ± 2.1 | Aerobic | 12 | 3 | 50 | 60-100% HRmax | 2.02 |
|  |  |  |  | EX (POST-M) | 37 | 53.3 ± 3 |  |  |  |  |  | 7.77 |
| Enrico et al. 2018 | Cohort pre-post | Chronic Kidney Disease | Clinically Stable Renal Transplant Recipients | EX | 30 | 38.6 ± 13.1 | Aerobic + Resistance | 52 | 3 | 60 | Moderate | 14.53 |
| Hollekim et al. 2014 | RCT | Cardiovascular Risk | Patients with T2DM | EX (MIE) | 17 | 54.7 ± 5.3 | Aerobic (MIE vs HIIT) | 12 | 3 | nr | nr | -1.80 |
|  |  |  |  | EX (HIIT) | 20 | 58.6 ± 5 |  |  |  | HIIT: 40 | HIIT: 90-95% HRmax | 5.81 |
| Hordern et al. 2009 | RCT | Cardiovascular Risk | Patients with T2DM | EX | 88 | 56.1 ± 11.7 | Aerobic + Resistance | 52 | 150min p/week | 150min p/week | Moderate to vigorous | 3.40 |
|  |  |  |  | CON | 88 | 55 ± 8.5 |  |  |  |  |  | 3.41 |
| Huang et al. 2019 | RCT | Healthy | Sedentary healthy males | EX (MICT) | 18 | 21.9 ± 0.6 | Aerobic (MICT vs HIIT) | 6 | 5 | 30 | MICT: 60% VO2max | -2.13 |
|  |  |  |  | EX (HIIT) | 18 | 21.4 ± 0.4 |  |  |  |  | HIIT: 80% VO2max ON, 40% VO2max OFF | -0.74 |
|  |  |  |  | CON | 18 | 22 ± 0.5 |  |  |  |  |  | -0.72 |
| Isbel et al. 2013 | RCT | Chronic Kidney Disease | Patients with moderate CKD | EX | 36 | 60.2 ± 9.7 | Aerobic + Resistance | 52 | nr | 60 | Moderate (11-13 RPE) | 2.17 |
|  |  |  |  | CON | 36 | 62 ± 8.4 |  |  |  |  |  | -6.67 |
| Jorgensen et al. 2017 | RCT | Cardiovascular Risk | Overweight patients with T2DM and Dysregulated HbA1c | EX (only) | 16 | 57 ± 10 | Aerobic + Resistance | 16 | 3 | 60 | Aerobic: 65-85% HRmax  Resistance: nr | 0.00 |
|  |  |  |  | EX (+liraglutide) | 16 | 57 ± 10 |  |  |  |  |  | -1.69 |
| Mahjoub et al. 2019 | RCT | Athletes | Endurance-trained men | EX (HIIT85) | 8 | 27 ± 7 | Aerobic (HIIT85 vs HIIT115) | 6 | 3 | HIIT85: 1-7 min bouts with 2:1 work: rest ratio until exhaustion | HIIT85: 85% maximal aerobic power | 1.57 |
|  |  |  |  | EX (HIIT115) | 9 | 27 ± 7 |  |  |  | HIIT115: 30s-1min bouts with 1:2 work: rest ratio until exhaustion | HIIT115: 115% maximal aerobic power | 1.02 |
| Malfatto et al. 2017 | RCT | Cardiovascular Disease | Patients following Acute MI | EX | 34 | 59 ± 10 | Aerobic + Calisthenics | 5 | 5 | 90 | Moderate to anaerobic threshold | 18.24 |
|  |  |  |  | CON | 21 | 64 ± 11 |  |  |  |  |  | 3.57 |
| McGregor et al. 2018 | Non-RCT | Cardiovascular Disease | Patients treated with PCI for acute MI. | EX | 15 | 57 ± 10.7 | Aerobic + Resistance | 10 | 2 | Aerobic: 25-40  Resistance: 20 | Aerobic: 60-80% VO2peak  Resistance: nr | -1.71 |
|  |  |  |  | CON | 16 | 54.1 ± 10.6 |  |  |  |  |  | 0.00 |
| Morville et al. 2018 | Cohort pre-post | Healthy | Healthy elderly recreational cyclists | EX | 6 | 61 ± 8 | Aerobic | 2 | 7 | 631 | 53% VO2max | 9.52 |
| O'Driscoll et al. 2018 | Randomised Crossover Study | Healthy | Physically inactive men | EX | 20 | 21 ± 1.7 | Aerobic Interval | 2 | 3 | 7.5 | 7.5% of body mass on watt bike | 3.99 |
|  |  |  |  | CON | 20 | 21 ± 1.7 |  |  |  |  |  | -0.35 |
| Ofstad et al. 2014 | RCT | Cardiovascular Risk | Patients with T2DM and ≥1 additional CV risk factor | EX | 50 | 58 ± 15 | Aerobic | 104 | 3 | 30 | nr | -10.53 |
|  |  |  |  | CON | 50 | 58 ± 12 |  |  |  |  |  | -6.33 |
| Orlandi et al. 2020 | Cohort pre-post | Chronic Kidney Disease | Kidney Transplant Recipients in a stable clinical condition | EX | 21 | 46.8 ± 12 | Aerobic + Resistance | 52 | 3 | 60 | 60-70% maximum effort | 25.14 |
| Oxborough et al. 2019 | RCT (Same data as Spence 2011) | Healthy | Healthy untrained males | EX (aerobic) | 10 | 28.4 ± 1.9 | Aerobic vs Resistance | 24 | 3 | 60 | nr | 0.00 |
|  |  |  |  | EX (resistance) | 13 | 26.6 ± 1.3 |  |  |  |  |  | 6.25 |
| Rojek et al. 2015 | Cohort pre-post | Healthy | Healthy amateur adults preparing for a triathlon | EX | 21 | 33 ± 6 | Aerobic + Resistance | 52 | >5 | Aerobic:  11.3hr p/week  Resistance: 1.3hr p/week | nr | -2.80 |
| Sacre et al. 2014 | Non-RCT | Cardiovascular Risk | T2DM patients >40 years old with asymptomatic subclinical diastolic dysfunction | EX | 24 | 59 ± 10 | Aerobic + Resistance | 24 | 2 | 75 | Moderate to vigorous | 20.67 |
|  |  |  |  | CON | 25 | 60 ±9 |  |  |  |  |  | 22.86 |
| Santoso et al. 2019 | Cohort pre-post | Cardiovascular Disease | Symptomatic heart failure with New York Heart Association class II–III | EX | 30 | 65.3 ± 6.2 | Aerobic | 4 | 3-5 | 90 | nr | 34.94 |
| Schmidt et al. 2013 | Non-RCT | Cardiovascular Risk | Sedentary men with T2DM. | EX | 12 | 50.6 ±7.1 | Aerobic | 24 | 2 | 60 | 82% HRmax | 8.97 |
|  |  |  |  | CON | 9 | 48.7 ± 9.2 |  |  |  |  |  | -10.29 |
| Schuster et al. 2012 | Cohort pre-post | Cardiovascular Risk | Obese men with BMI between 30 and 40 kg/m2 | EX | 10 | 52.4 ±3.2 | Aerobic | 8 | 3 | 45 | HR equivalent of 50% VO2max | 9.43 |
|  |  |  |  | CON | 14 | 46.9 ±2.1 |  |  |  |  |  | nr |
| Serrano-Ferrer et al. 2016 | RCT | Cardiovascular Risk | Adults with metabolic syndrome | EX (rE) | 27 | 57 ± 4 | Aerobic + Resistance | 24 | 4-5 | 90 | rE:  30% 1RM, 70% VO2peak | 14.97 |
|  |  |  |  | EX (re) | 32 | 59 ± 5 |  |  |  |  | re:  30% 1RM, 30% VO2peak | 14.81 |
|  |  |  |  | EX (Re) | 28 | 61 ± 5 |  |  |  |  | Re:  70% 1RM, 30% VO2peak | 11.31 |
|  |  |  |  | CON | 44 | 58 ±4 |  |  |  |  |  | 13.94 |
| Spence et al. 2011 | RCT (Same data as Oxborough 2019) | Healthy | Young healthy males | EX (aerobic) | 10 | 28.4 ±1.9 | Aerobic vs Resistance | 24 | 3 | 60 | nr | 3.39 |
|  |  |  |  | EX (resistance) | 13 | 26.6 + 1.3 |  |  |  |  |  | 2.83 |
| Traschel et al. 2019 | RCT | Cardiovascular Disease | Patients with Acute MI in last 6 weeks | EX | 9 | 60 ± 10 | Aerobic Interval + Resistance | 12 | 2 | HIIT: 20  Resistance: 20 | 15 RPE | 5.85 |
|  |  |  |  | CON | 10 | 57 ± 13 |  |  |  |  |  | 9.94 |
| VanDeHeyening et al. 2018 | RCT | Cardiovascular Disease | Patients referred for CR either following angiographically documented CAD or AMI with PCI or CABG in the last 4-12 weeks. | EX (AIT) | 97 | 57 ± 9 | Aerobic (AIT vs ACT) | 12 | 3 | AIT: 38 | AIT:  85-95% peak HR (work)/50-70% peak HR (rest) | 4.85 |
|  |  |  |  | EX (ACT) | 97 | 60 ± 9 |  |  |  | ACT: 47 | ACT:  70-75% peak HR | 0.55 |
| Weiner et al. 2010 | Cohort pre-post | Athletes | Rowing athletes from Harvard University Athletics Department | EX | 15 | 18.6 ± 0.5 | Aerobic + Resistance | 12 | >5 | 13.6 +- 0.9 hrs. p/week of organised training (aerobic  12.7 +- 0.7 h/wk; and strength 1.0 +- 0.9 h/wk | nr | 8.93 |
| Xu et al. 2016 | RCT | Cardiovascular | Adults with Acute MI diagnosis, suitable for the CR program | EX | 26 | 55.8 ± 9.7 | Aerobic | 4 | 7 | 60-90 | 60% MaxHR and <6 RPE | 23.97 |
|  |  |  |  | CON | 26 | 55.5 ± 8.9 |  |  |  |  |  | 0.00 |
| Zilinski et al. 2015 | Cohort pre-post | Healthy | Healthy men | EX | 45 | 47.7 ± 7.4 | Aerobic | 18 | 4-5 | 4.0+-2.2 hrs. p/week | nr | -0.54 |

Abbreviations: RCT - randomised control trial; Non-RCT - non-randomised control trial; MI - myocardial infarction; HFpEF - heart failure preserved ejection fraction; CAD - coronary heart disease; T2DM - type 2 diabetes; CKD - chronic kidney disease; CV - cardiovascular; BMI - body mass index; CR - cardiac rehabilitation; PCI - percutaneous coronary intervention; CABG - coronary artery bypass graft; EX - exercise; CON - control; HIIT - high intensity interval training; MICT - moderate intensity continuous training; HR-LL - high repetitions, low load; LR-HL - low repetitions, high load; LP - linear progression; NLP - non-linear progression; PRE-M - pre-menopause; POST-M - post-menopause; MIE - moderate intensity exercise; AIT - aerobic interval training; ACT - aerobic continuous training; *n* - number of participants; yrs - years; nr - not reported; wk/s - week/s; p/week - per week; mins - minutes; hrs - hours; h/wk - hours per week; %, percent; RPE - rating of perceived exertion; HR - heart rate; RM - repetition maximum; HRmax - heart rate maximum; VO2max - maximum oxygen consumption; VO2peak - peak oxygen consumption.

**Table 3.** Methods and reporting of strain analysis.

| **Author** | **Views used for strain** | **Excluded due to image quality** | **Machine used** | **Analysis software used** | **Reliability measures** |
| --- | --- | --- | --- | --- | --- |
| Acar et al 2015 | Apical 4ch view | Not reported | GE Vivid 7 | Not reported | r: 0.867 |
| Acar et al 2015b | Apical 4ch view | Not reported | GE Vivid 7 | Not reported | r: 0.867 |
| Aksakal et al 2013 | Apical 4, 2 and long axis views | Not reported | GE Vivid 7 | GE EchoPAC v6.1 | ICC: >0/75 |
| Anderson et al 2014 | Not reported | Not reported | GE Vivid 9 | GE EchoPAC BT11 | <5% |
| Angaldi et al 2017 | Not reported | Not reported | Siemens | Siemens Syngo US workstation | Not reported |
| Au et al 2019 | Not reported | 6/46 excluded | GE Vivid q | GE EchoPAC V110.0.2 | ICC: >94% |
| Boidin et al 2020 | Apical 4, 2 and long axis views | Not reported | GE Vivid 9 | GE EchoPAC (version not reported) | Not reported |
| Cadeddu et al 2016 | Apical 4 and 2 chamber views | Not reported | Toshiba | Toshiba (version not reported) | Not reported |
| D’Ascenzi et al 2014 | Not reported | Not reported | GE Vivid 7 | GE EchoPAC v112.1.1 | Not reported |
| D’Ascenzi et al 2015 | Apical 4, 2 and long axis views | Not reported | GE Vivid 9 | GE EchoPAC v.112 | Not reported |
| D’Ascenzi et al 2016 | Apical 4 chamber | Not reported | GE Vivid 9 | GE EchoPAC v.112 | Not reported |
| D’Silva et al 2020 | Not reported | Not reported | GE Vivid E9 | Ge EchoPAC v.113 | ICC: >0.75 |
| Egelund et al 2017 | Not reported | 2/83 “not assessed by echocardiography for logistic reasons” | GE Vivid 9 | GE EchoPAC BT13.0 | Variation: 0% - 15.2%. |
| Enrico et al 2018 | Apical 4, 2 and long axis views | Not reported | MyLab 50 | XStrain - ESAOTE | Not reported |
| Hollekim et al 2014 | Not reported | Not reported | Not reported | Not reported | Not reported |
| Horden et al 2009 | Tissue Doppler Imaging strain from apical 4, 2 and long axis views | Not reported | GE Vivid 7 | GE EchoPAC (version not reported) | k = 0.8 |
| Huang et al 2019 | Apical 4, 2 and long axis views | All subjects had suitable images | Siemens ACUSON SC2000 | Siemens VVI | Not reported |
| Isbel et al 2013 | Not reported | Not reported | GE Vivid 7 | Not reported | Not reported |
| Jorgensen et al 2017 | Apical 4, 2 and long axis views | Not reported | GE Vivid 7 & 9 | GE EchoPAC BT13 | Not reported |
| Mahjoub et al 2019 | Apical 4, 2 and long axis views | Not reported | Phillips IE33 | TomTec 2D Cardiac Performance Analysis | ICC: 0.91 |
| Malfatto et al 2017 | Apical 4, 2 and long axis views | Not reported | GE Vivid 7 & 9 | GE EchoPAC (version not reported) | Not reported |
| McGregor et al 2018 | Apical 4ch view | 5/40 excluded | GE Vivid 7 | GE EchoPAC v7.0 | Not reported |
| Morville et al 2018 | Apical 4, 2 and long axis views | Not reported | GE Vivid E9 or GE S6 | GE EchoPAC v11.2 | Not reported |
| O’Driscoll et al 2018 | Apical 4, 2 and long axis views | Not reported | GE Vivid q | Not reported | Not reported |
| Ofstad et al 2014 | Tissue Doppler Imaging strain from apical 4 and 2 chamber views | Not reported | GE Vivid 5 or 7 | Not reported | “good” |
| Orlandi et al 2020 | Apical 4, 2 and long axis views | Not reported | MyLab 50 | XStrain - ESAOTE | Not reported |
| Oxborough et al 2019 | Apical 4 chamber view | Not reported | GE Vivid 1 | GE EchoPAC (version not reported) | “good” |
| Rojek et al 2015 | Apical 4, 2 and long axis views | Not reported | GE Vivid E9 | GE EchoPAC (version not reported) | -0.3% |
| Sacre et al 2014 | Tissue Doppler Imaging strain from apical 4 and 2 chamber views | Not reported | GE Vivid 7 | GE EchoPAC (version not reported) | Not reported |
| Santoso et al 2019 | Apical 4ch view | Not reported | GE Vivid 7 | Not reported | 8.6% |
| Schmidt et al 2013 | Not reported | Not reported | GE Vivid 9 | GE EchoPAC BT11.0 | Not reported |
| Schuster et al 2012 | Apical 4ch view | Not reported | GE Vivid 7 | GE EchoPAC v6.0 | <8% |
| Serrano-Ferrer et al 2016 | Apical 4ch view | Not reported | Not reported | Siemens VVI | Pc: 0.90 |
| Spence et al 2011 | Apical 4ch view | Not reported | GE Vivid 1 | GE EchoPAC (version not reported) | Not reported |
| Traschel et al 2019 | Apical 4, 2 and long axis views | Not reported | GE Vivid 9 | GE EchoPAC (version not reported) | Not reported |
| VanDe Heyning et al 2018 | Apical 4, 2 and long axis views | Not reported | GE Vivid 7 or 9 | GE EchoPAC (version not reported) | Not reported |
| Weiner et al 2010 | Not reported | All subjects had suitable images | GE Vivid 1 | GE EchoPAC V7 | r^2^ = >0.9 |
| Xu et al 2016 | Global strain from 3D dataset | 6/70 excluded | GE Vivid E9 | GE EchoPAC BT11.1.0 | ICC: 0.95 |
| Zilinski et al 2015 | Not reported | Not reported | GE Vivid 1 | GE EchoPAC v6.5 | Not reported |

Figure 9. Effect of exercise intervention length on LVGLS using exercise data from randomised control trials, non-randomised control trials, randomised cross over trials and single group pre-post studies.

Abbreviations: AMI – acute myocardial infarction; HIIT – high intensity interval training; HFpEF – heart failure preserved ejection fraction; MICT – moderate intensity continuous training; HR-LL – high repetitions-low load; LR-HL – low repetitions, high load; CAD – coronary artery disease; IR – insulin resistance; T2D – type 2 diabetes; ACT – aerobic continuous training; AIT – aerobic interval training; HTN – hypertension; RTR – renal transplant recipient; CKD – chronic kidney disease; MetS – metabolic syndrome; CI – confidence intervals.

Figure 10. Effect of exercise modality on LVGLS using exercise data from randomised control trials, non-randomised control trials, randomised cross over trials and single group pre-post studies – Comparison between aerobic training only vs a combination of aerobic and resistance training.

Abbreviations: AMI – acute myocardial infarction; HTN – hypertension; HIIT – high intensity interval training; HFpEF – heart failure preserved ejection fraction; MICT – moderate intensity continuous training; IR – insulin resistance; T2D – type 2 diabetes; ACT – aerobic continuous training; AIT – aerobic interval training; CAD – coronary artery disease; RTR – renal transplant recipient; CKD – chronic kidney disease; MetS – metabolic syndrome; CI – confidence intervals.

Figure 11. Effect of exercise modality on LVGLS using exercise data from randomised control trials, non-randomised control trials, randomised cross over trials and single group pre-post studies – Comparison between aerobic continuous training vs aerobic interval training.

Abbreviations: AMI – acute myocardial infarction; MICT – moderate intensity continuous training; HFpEF – heart failure preserved ejection fraction; IR – insulin resistance; MIE – moderate intensity exercise; T2D – type 2 diabetes; ACT – aerobic continuous training; CAD – coronary artery disease; HTN – hypertension; HIIT – high intensity interval training; AIT – aerobic interval training; CI – confidence intervals.

Figure 12. Randomised control trials, non-randomised control trials and randomised crossover trials investigating the effect of exercise on LVGLS in healthy populations alone.

Abbreviations: HR-LL – high repetitions-low load; LR-HL – low repetitions, high load; MICT – moderate intensity continuous training; HIIT – high intensity interval training; N – number of participants in each group; SD – standard deviation; CI – confidence intervals.

Figure 13. Exercise data from randomised control trials, non-randomised control trials, randomised cross over trials and single group pre-post studies investigating the effect of exercise on LVGLS in cardiovascular disease populations alone.

Abbreviations: AMI – acute myocardial infarction; HIIT – high intensity interval training; HFpEF – heart failure preserved ejection fraction; MICT – moderate intensity continuous training; CAD – coronary artery disease; ACT – aerobic continuous training; AIT – aerobic interval training; CI – confidence intervals.

Figure 14. Exercise data from randomised control trials, non-randomised control trials, randomised cross over trials and single group pre-post studies investigating the effect of exercise on LVGLS in populations with cardiovascular risk factors alone.

Abbreviations: HTN – hypertension; IR – insulin resistance; MICT – moderate intensity continuous training; T2D – type 2 diabetes; HIIT – high intensity interval training; EX – exercise; MetS – metabolic syndrome; CI – confidence intervals.

Figure 15. Single group pre-post studies investigating the effect of exercise on LVGLS in chronic kidney disease populations alone.

Abbreviations: CKD – chronic kidney disease; RTR – renal transplant recipient; CI – confidence intervals.

Figure 16. Exercise data from randomised control trials, non-randomised control trials, randomised cross over trials and single group pre-post studies investigating the effect of exercise on LVGLS in healthy populations alone.

Abbreviations: HR-LL – high repetitions-low load; LR-HL – low repetitions, high load; MICT – moderate intensity continuous training; HIIT – high intensity interval training; CI – confidence intervals.

Figure 17. Single group pre-post studies investigating the effect of exercise on LVGLS in athletic populations alone.

Abbreviations: HIIT85% - high intensity interval training at 85% maximum workload; HIIT115% - high intensity interval training at 115% maximum workload; CI – confidence intervals.

Table 4. Summary of meta-analysis outcomes per health category.

| **Population** | **n** | **SMD** | **95% CI** | **p** | **I^2^** |
| --- | --- | --- | --- | --- | --- |
| *Primary Analysis* |  |  |  |  |  |
| Cardiovascular Disease | 4 | 0.59 (i) | 0.16-1.02 | 0.01^a^ | 40.12% |
| Cardiovascular Risk | 5 | 0.07 (i) | -0.15-0.29 | 0.56 | 0.00% |
| Healthy | 4 | -0.20 (c) | -0.73-0.33 | 0.45 | 59.08% |
| *Secondary Analysis* |  |  |  |  |  |
| Cardiovascular Disease | 7 | 0.26 | 0.07-0.46 | 0.01^a^ | 73.87% |
| Cardiovascular Risk | 10 | 0.54 | 0.15-0.93 | 0.01^a^ | 94.46% |
| Chronic Kidney Disease | 3 | 0.65 | 0.03-1.28 | 0.04^a^ | 93.66% |
| Healthy | 14 | 0.15 | -0.01-0.31 | 0.06 | 78.09% |
| Athletic | 6 | 0.30 | 0.20-0.41 | <0.001^a^ | 0.00% |

Abbreviations: n – number of studies included in analysis; SMD – standardized mean difference; CI – confidence intervals; p – significance; I^2^ – heterogeneity; % - percentage.
^a^ Significant difference pre-post exercise (p=<0.05). (i) Effect in favour of intervention group (exercise). (c) Effect in favour of control group.

Table 5. Summary of exploratory meta-analyses using pre-post study data.

| **Exploratory Analyses** | **n** | **SMD** | **95% CI** | **p** | **I^2^** |
| --- | --- | --- | --- | --- | --- |
| *Intervention Lengths (weeks)* |  |  |  |  |  |
| 0-11.9 | 10 | 0.38 | 0.08-0.69 | 0.01^a^ | 83.43% |
| 12-23.9 | 17 | 0.17 | 0.05-0.29 | <0.001^a^ | 74.91% |
| 24 + | 13 | 0.50 | 0.23-0.76 | <0.001^a^ | 94.20% |
| *Exercise Modality (Aerobic vs Combination)* |  |  |  |  |  |
| Aerobic Only | 24 | 0.29 | 0.15-0.43 | <0.001^a^ | 81.63% |
| Combination of AT and RT | 13 | 0.42 | 0.17-0.66 | <0.001^a^ | 94.31% |
| *Exercise Modality (Aerobic Continuous vs Aerobic Interval)* |  |  |  |  |  |
| Aerobic Continuous | 14 | 0.25 | 0.03-0.47 | 0.01^a^ | 87.39% |
| Aerobic Interval | 11 | 0.34 | 0.18-0.50 | <0.001^a^ | 65.48% |

Abbreviations: + - plus; AT – aerobic training; RT – resistance training; n – number of studies included in analysis; SMD – standardized mean difference; CI – confidence intervals; p – significance; I^2^ – heterogeneity; % - percentage; < - less than.
^a^ Significant difference pre-post exercise (p=<0.05).

**Reference List:**

1. Acar RD, Bulut M, Ergun S, Yesin M, Akcakoyun M. Evaluation of the effect of cardiac rehabilitation on left atrial and left ventricular function and its relationship with changes in arterial stiffness in patients with acute myocardial infarction. Echocardiography. 2015;32(3):443-7.

2. Acar RD, Bulut M, ErgÜN S, YesİN M, Kalkan ME, AkÇAkoyun M. Assessment of the Left Ventricular Systolic Function of Patients with Acute Myocardial Infarction after Cardiac Rehabilitation by Using Two Dimensional Echocardiography. / Akut Miyokard İnfarktüsü Geçirmiş Hastalarda Kardiyak Rehabilitasyon Sonrası Sol Ventrikül Sistolik Fonksiyonunun İki Boyutlu Ekokardiyografi Benek Takibi Analiz Yöntemi ile Değerlendirilmesi. Turkish Journal of Physical Medicine & Rehabilitation / Turkiye Fiziksel Tip ve Rehabilitasyon Dergisi. 2015;61(3):211-5.

3. Aksakal E, Kurt M, Ozturk ME, Tanboga IH, Kaya A, Nacar T et al. The effect of incremental endurance exercise training on left ventricular mechanics: a prospective observational deformation imaging study. Anadolu Kardiyoloji Dergisi. 2013;13(5):432-8.

4. Andersen LJ, Randers M, Hansen PR, Hornstrup T, Schmidt JF, Dvorak J et al. Structural and functional cardiac adaptations to 6 months of football training in untrained hypertensive men. Scandinavian journal of medicine & science in sports. 2014;24:27-35.

5. Angadi SS, Jarrett CL, Sherif M, Gaesser GA, Mookadam F. The effect of exercise training on biventricular myocardial strain in heart failure with preserved ejection fraction. ESC heart failure. 2017;4(3):356-9.

6. Au JS, Oikawa SY, Morton RW, Phillips SM, MacDonald MJ, Stöhr EJ. Unaltered left ventricular mechanics and remodelling after 12 weeks of resistance exercise training–a longitudinal study in men. Applied Physiology, Nutrition, and Metabolism. 2019;44(8):820-6.

7. Boidin M, David LP, Trachsel LD, Gayda M, Tremblay J, Lalonge J et al. Impact of Two Different Aerobic Periodization Training Protocols on Left Ventricular Function in Patients with Stable Coronary Artery Disease: An Exploratory Study. Applied physiology, nutrition, and metabolism = Physiologie appliquee, nutrition et metabolisme. 2020;27.

8. Cadeddu C, Nocco S, Cugusi L, Deidda M, Fabio O, Bandino S et al. Effects of Metformin and Exercise Training, Alone or in Combination, on Cardiac Function in Individuals with Insulin Resistance. Cardiology and Therapy. 2016;5(1):63-73.

9. D’Silva A, Bhuva AN, Van Zalen J, Bastiaenen R, Abdel-Gadir A, Jones S et al. Cardiovascular remodeling experienced by real-world, unsupervised, young novice marathon runners. Frontiers in physiology. 2020;11:232.

10. D'Ascenzi F, Pelliccia A, Alvino F, Solari M, Loffreno A, Cameli M et al. Effects of training on LV strain in competitive athletes. Heart. 2015;101(22):1834-9.

11. D'Ascenzi F, Pelliccia A, Corrado D, Cameli M, Curci V, Alvino F et al. Right ventricular remodelling induced by exercise training in competitive athletes. European heart journal cardiovascular Imaging. 2016;17(3):301-7.

12. D'Ascenzi F, Pelliccia A, Natali BM, Zaca V, Cameli M, Alvino F et al. Morphological and functional adaptation of left and right atria induced by training in highly trained female athletes. Circulation Cardiovascular imaging. 2014;7(2):222-9.

13. Egelund J, Jørgensen PG, Mandrup CM, Fritz‐Hansen T, Stallknecht B, Bangsbo J et al. Cardiac adaptations to high‐intensity aerobic training in premenopausal and recent postmenopausal women: the copenhagen women study. Journal of the American Heart Association. 2017;6(8):e005469.

14. Enrico M, Klika R, Ingletto C, Mascherini G, Pedrizzetti G, Stefani L. Changes in global longitudinal strain in renal transplant recipients following 12 months of exercise. Internal & Emergency Medicine. 2018;13(5):805-9.

15. Hollekim-Strand SM, Bjørgaas MR, Albrektsen G, Tjønna AE, Wisløff U, Ingul CB. High-intensity interval exercise effectively improves cardiac function in patients with type 2 diabetes mellitus and diastolic dysfunction: a randomized controlled trial. Journal of the American College of Cardiology. 2014;64(16):1758-60.

16. Hordern MD, Coombes JS, Cooney LM, Jeffriess L, Prins JB, Marwick TH. Effects of exercise intervention on myocardial function in type 2 diabetes. Heart. 2009;95(16):1343-9.

17. Huang YC, Tsai HH, Fu TC, Hsu CC, Wang JS. High-Intensity Interval Training Improves Left Ventricular Contractile Function. Medicine and Science in Sports and Exercise. 2019;51(7):1420-8. doi:10.1249/MSS.0000000000001931.

18. Isbel N, Howden E, Leano R, Petchey W, Coombes J. Exercise and lifestyle intervention in chronic kidney disease: Effects on cardiovascular function. Nephrology. 2013;18:27.

19. Jørgensen PG, Jensen MT, Mensberg P, Storgaard H, Nyby S, Jensen JS et al. Effect of exercise combined with glucagon‐like peptide‐1 receptor agonist treatment on cardiac function: A randomized double‐blind placebo‐controlled clinical trial. Diabetes, Obesity and Metabolism. 2017;19(7):1040-4.

20. Mahjoub H, Le Blanc O, Paquette M, Imhoff S, Labrecque L, Drapeau A et al. Cardiac remodeling after six weeks of high-intensity interval training to exhaustion in endurance-trained men. American Journal of Physiology - Heart & Circulatory Physiology. 2019;317(4):H685-H94.

21. Malfatto G, Revera M, Branzi G, Ciambellotti F, Giglio A, Blengino S et al. A brief period of intensive cardiac rehabilitation improves global longitudinal strain and diastolic function after a first uncomplicated myocardial infarction. Acta Cardiologica. 2017;72(3):284-91.

22. McGregor G, Stöhr EJ, Oxborough D, Kimani P, Shave R. Effect of exercise training on left ventricular mechanics after acute myocardial infarction–an exploratory study. Annals of Physical and Rehabilitation Medicine. 2018;61(3):119-24. doi:10.1016/j.rehab.2018.01.003.

23. Morville T, Rosenkilde M, Mattsson N, Dela F, Helge JW, Rasmusen HK. 2706 km cycling in 2 weeks: effects on cardiac function in 6 elderly male athletes. Physician and Sportsmedicine. 2018;46(3):263-8. doi:10.1080/00913847.2018.1477403.

24. O'Driscoll JM, Wright SM, Taylor KA, Coleman DA, Sharma R, Wiles JD. Cardiac autonomic and left ventricular mechanics following high intensity interval training: a randomized crossover controlled study. Journal of Applied Physiology. 2018;125(4):1030-40.

25. Ofstad AP, Johansen OE, Gullestad L, Birkeland KI, Orvik E, Fagerland MW et al. Neutral impact on systolic and diastolic cardiac function of 2 years of intensified multi-intervention in type 2 diabetes: the randomized controlled Asker and Bærum Cardiovascular Diabetes (ABCD) study. American heart journal. 2014;168(3):280-8. e2.

26. Orlandi G, Sofi F, Moscarelli L, Cirami L, Mancini S, Stefani L. Exercise prescription in renal transplant recipients: From sports medicine toward multidisciplinary aspects: A pilot study. Journal of Functional Morphology and Kinesiology. 2020;5(1). doi:10.3390/jfmk5010010.

27. Oxborough DL, Spence A, George KP, Van Oorschot F, Thijssen DHT, Green DJ. Impact of 24 weeks of supervised endurance versus resistance exercise training on left ventricular mechanics in healthy untrained humans. Journal of Applied Physiology. 2019;126(4):1095-102.

28. Rojek A, Bialy D, Przewlocka-Kosmala M, Negrusz-Kawecka M, Mysiak A, Kosmala W. Biventricular response of the heart to endurance exercise training in previously untrained subjects. Echocardiography. 2015;32(5):779-86.

29. Sacre JW, Jellis CL, Jenkins C, Haluska BA, Baumert M, Coombes JS et al. A six-month exercise intervention in subclinical diabetic heart disease: effects on exercise capacity, autonomic and myocardial function. Metabolism. 2014;63(9):1104-14.

30. Santoso A, Purwowiyoto SL, Purwowiyoto BS, Soesanto AM. Exercise Training Improved Longitudinal Intrinsic Left Ventricle Function in Heart Failure with Preserved Ejection Fraction. International Journal of Angiology. 2019;28(1):44-9. doi:10.1055/s-0038-1676836.

31. Schmidt JF, Andersen TR, Horton J, Brix J, Tarnow L, Krustrup P et al. Soccer training improves cardiac function in men with type 2 diabetes. Medicine & Science in Sports & Exercise. 2013;45(12):2223-33.

32. Schuster I, Vinet A, Karpoff L, Startun A, Jourdan N, Dauzat M et al. Diastolic dysfunction and intraventricular dyssynchrony are restored by low intensity exercise training in obese men. Obesity. 2012;20(1):134-40.

33. Serrano-Ferrer J, Crendal E, Walther G, Vinet A, Dutheil F, Naughton G et al. Effects of lifestyle intervention on left ventricular regional myocardial function in metabolic syndrome patients from the RESOLVE randomized trial. Metabolism: Clinical & Experimental. 2016;65(9):1350-60.

34. Spence AL, Naylor LH, Carter HH, Buck CL, Dembo L, Murray CP et al. A prospective randomised longitudinal MRI study of left ventricular adaptation to endurance and resistance exercise training in humans. Journal of Physiology. 2011;589(Pt 22):5443-52.

35. Trachsel LD, David LP, Gayda M, Henri C, Hayami D, Thorin‐Trescases N et al. The impact of high‐intensity interval training on ventricular remodeling in patients with a recent acute myocardial infarction—A randomized training intervention pilot study. Clinical cardiology. 2019;42(12):1222-31.

36. Van De Heyning CM, De Maeyer C, Pattyn N, Beckers PJ, Cornelissen VA, Goetschalckx K et al. Impact of aerobic interval training and continuous training on left ventricular geometry and function: a SAINTEX-CAD substudy. International Journal of Cardiology. 2018;257:193-8.

37. Weiner RB, Hutter AM, Jr., Wang F, Kim J, Weyman AE, Wood MJ et al. The impact of endurance exercise training on left ventricular torsion. Jacc: Cardiovascular Imaging. 2010;3(10):1001-9.

38. Xu L, Cai Z, Xiong M, Li Y, Li G, Deng Y et al. Efficacy of an early home-based cardiac rehabilitation program for patients after acute myocardial infarction: A three-dimensional speckle tracking echocardiography randomized trial. Medicine. 2016;95(52):e5638.

39. Zilinski JL, Contursi ME, Isaacs SK, Deluca JR, Lewis GD, Weiner RB et al. Myocardial adaptations to recreational marathon training among middle-aged men. Circulation: Cardiovascular Imaging. 2015;8(2):e002487.
