# Supplementary material for: The effect of exercise on left ventricular global longitudinal strain
Source: Eur J Appl Physiol. 2022 Mar 16;122(6):1397–408. doi: 10.1007/s00421-022-04931-5 (PMC9132819; doi:10.1007/s00421-022-04931-5)
Supplement: Supplementary file 2 — Supplementary file2 (DOCX 23 KB) [file 421_2022_4931_MOESM2_ESM.docx]

**Supplementary Material 2**

**2.1. Selection criteria**

Studies performed on humans and published in the English language from 2000 onwards were included in this review. Only studies published after 2000 were included due to the advancement of measurement techniques for GLS in the last 20 years. All exercise modalities (aerobic, resistance, combination) were included. Participants taking cardioprotective medications were included. Randomised control trials (RCTs), non-randomised control trials (N-RCTs), cohort, cross over and observational studies were all included. Participants taking cardiotoxic and/or cardiovascular limiting medications (i.e. chemotherapy) were excluded as it is well established that these impact upon GLS negatively (Yang et al. 2018). To provide a full representation of the available literature, papers were not excluded if they failed to report sufficient statistics for estimating effect sizes, did not justify the selected sample size, or on the basis of risk of bias assessment. Abstracts, reviews, meta-analyses, and other secondary sources were also excluded.

**2.2. Study selection and data extraction**

Two authors (JM and HB) independently conducted all database searches, abstract screening, and full text review. References identified through database searches were imported into Covidence Systematic Review Software (Veritas Health Innovation, Melbourne, Australia). Abstracts were independently reviewed for initial eligibility. Where eligibility was met or could not be determined, full text versions of studies were accessed and reviewed. Discrepancies were resolved by discussion between all authors.

A data extraction template (Microsoft Excel Version 16.10, Redmond, Washington, USA) was developed a priori and piloted on two randomly selected studies meeting eligibility criteria. One author (JM) independently extracted all data. Extraction was then cross referenced by (HB), with any discrepancies discussed and resolved.

Data were extracted across the following domains:
Publication demographics: title, authors, year of publication
Study characteristics: study design, measurement time points, significance level, population (age, sex, sample size, comorbidities, medications), health category (healthy, athletic, cardiovascular disease, cardiovascular risk, chronic kidney disease).
Training protocols: exercise modality (aerobic continuous, aerobic interval, resistance or combination), frequency (sessions per week), intensity, intervention duration (length of intervention) and session duration (length of each session).
Outcome Measures: left ventricular global longitudinal strain (LVGLS).

Studies allocated to the ‘healthy’ category included participants with no reported chronic health conditions, taking no mediations and had no contraindications to exercise. Studies allocated to the ‘cardiovascular disease’ category included participants diagnosed with and medicated for any form of overt CV disease, including, coronary artery disease and heart failure or any participants post CV event (e.g. post myocardial infarction, hospitalization for heart failure). Studies allocated to the ‘cardiovascular risk’ category included participants diagnosed with or medicated for any CV disease risk factors including hypertension, metabolic syndrome, impaired glucose tolerance, insulin resistance, type 1 or 2 diabetes, or obesity. Studies allocated to the ‘athletes’ category included participants who competed in team sports at a national or international level, were enrolled in athletic scholarship programs, or completed >5 hours of aerobic or resistance training per week prior to the participating in the study. Studies allocated to the ‘chronic kidney disease’ category, included participants following renal transplant, or diagnosed with chronic kidney disease.

**2.3. Data analysis**

Percentage change in LVGLS from baseline was calculated for each individual study using the following formula: [(post LVGLS – pre LVGLS)/pre LVGLS] x 100. These results were then summarised descriptively.

Primary meta-analyses:
Standardized mean difference (SMD) between intervention and control groups at post-treatment weas calculated using Cohen’s d statistic [25] with 95% confidence intervals (CI). SMD was categorised as small (0.2), medium (0.5) and large (0.8) [25]. Heterogeneity was calculated using the I^2^ statistic. I^2^ values of 25%, 50% and 75% were considered to represent low, moderate, and high heterogeneity, respectively.

Secondary meta-analyses:
SMD between pre and post data in the intervention group was calculated using Cohen’s d statistic with 95% CI’s. The effect size and variance of each individual study was calculated using pre- and post-intervention means, the pre-intervention SD, and the pre-post correlation coefficient (r) using the package ‘metafor’ in R (v3.5.0, Core Team, Vienna, Austria). No studies reported the actual value of r, so it was estimated using the following formula:

$$r={{{((SD}_{pre})}^{2}+{{(SD}_{post})}^{2}-{{(SD}_{change})}^{2})}/{(2x\left( {SD}_{pre}*{SD}_{post} \right))}$$

In studies that did not report the SD of the change, the mean of the calculated correlation coefficients was used. The calculated effect size and variance for each individual study was then inputted into Stata to perform secondary meta-analyses.

Sub-group exploratory meta-analyses:
Exploratory meta-analyses were performed to explore the relationship between intervention lengths, exercise modalities and LVGLS, using the same data as per the secondary meta-analyses. Studies were not divided into health categories when performing these exploratory analyses, but rather studies were categorised based on exercise intervention characteristics (i.e., duration, modality). Intervention durations were categorised into three groups: 0-11.9 weeks, 12-23.9 weeks, and 24 weeks or greater for analysis. Exercise modalities compared were aerobic training only (including continuous, interval or both) vs a combination of aerobic and resistance training, and aerobic continuous training vs aerobic interval training. Meta-regression analyses were performed to determine whether the observed SMD’s differed between intervention length or exercise modality.

**2.4. Quality assessment**

Quality assessment of manuscript data was performed independently by two authors (JM and HB). Any discrepancies were resolved by a third author (RP). The QUADAS-2 tool [27] was used to evaluate the risk of bias associated with the population, methodology, analysis and reporting of each included study. Explicit instructions on how to use the tool can be found via the following link: [QUADAS-2 Tool](https://www.bristol.ac.uk/population-health-sciences/projects/quadas/quadas-2/). In brief, the tool consists of four key domains: patient selection, index test, reference standard and flow and timing. Each domain is assessed in terms of risk of bias, with only the first three domains assessed in terms of concerns regarding applicability to the research question. For each individual study, a rating of “low”, “high” or “unclear” was given in each domain. The ratings of each domain are then displayed, with the overall quality of included studies subjectively reported.
